# The Progamic Phase in High-Mountain Plants: From Pollination to Fertilization in the Cold 

**DOI:** 10.3390/plants2030354

**Published:** 2013-06-25

**Authors:** Gerlinde Steinacher, Johanna Wagner

**Affiliations:** Institute of Botany, Faculty of Biology, University of Innsbruck, Sternwartestrasse 15, Innsbruck 6020, Austria; E-Mail: gerlinde.steinacher@uibk.ac.at

**Keywords:** alpine plants, fertilization, plant reproduction, pollen germination, pollen tube growth, low temperature, cold snap

## Abstract

In high-mountains, cold spells can occur at any time during the growing season and plants may be covered with snow for several days. This raises the question to what extent sexual processes are impaired by low temperatures. We tested pollen performance and fertilization capacity of high-mountain species with different elevational distribution in the European Alps (*Cerastium uniflorum*, *Gentianella germanica*, *Ranunculus glacialis*, *R. alpestris*, *Saxifraga bryoides*, *S. caesia*, *S. moschata*) during simulated cold snaps in the laboratory. Plants were exposed to 0 °C (the temperature below the snow) for 12, 36, 60 and 84 h. In *S. caesia*, the experiment was verified *in situ* during a cold snap. Sexual processes coped well with large temperature differences and remained functional at near-freezing temperatures for a few days. During the cooling-down phase a high percentage (67–97%) of pollen grains germinated and grew tubes into the style. At zero degrees, tube growth continued slowly both in the laboratory and *in situ* below the snow. Fertilization occurred in up to 100% of flowers in the nival species and in *G. germanica*, but was strongly delayed or absent in the alpine species. During rewarming, fertilization continued. Overall, progamic processes in high-mountain plants appear fairly robust toward weather extremes increasing the probability of successful reproduction.

## 1. Introduction

Anthesis is one of the most critical phases in the life cycle of a plant and extremely vulnerable to unfavorable climatic conditions. The different male and female processes during the progamic phase (*i.e.*, the phase between pollination and fertilization) are strongly affected by temperature and can only be maintained within a certain temperature range [1,2,3,4]. Temperatures that are too low as well as those that are too high result in reduced reproductive success or complete reproductive failure [5]. Temperature limits for sexual processes vary among species and genotypes according to their adaptation to the temperature climate in the respective habitat [6,7,8,9,10,11]. In most lowland plants of the temperate zone, pollen germination and tube growth proceed optimally between 20 and 30 °C, but are drastically reduced below 10 °C [12,13,14,15,16] often leading to poor or no seed set [17,18]. Reports about pollen performance in plants from cooler habitats are rare. McKee and Richards [7] found tube growth in different *Primula* species to be most efficient between 15 and 26 °C, but still sufficient at 6 °C to give rise to seeds. 

High-mountain plants experience particularly wide temperature fluctuations during the growing season. On clear days, flowers may reach maximum temperatures of 25–30 °C [19,20], partly because of warming under heliotropism [21,22,23,24]. When cloudy and during the night, plants largely adopt the temperature of the free air. Night-time temperatures drop to about 5 °C in the alpine zone and to near zero in the nival zone [25,26]. In clear weather, plants may even cool down below the air temperature due to night-time radiation [27]. The constant switch between warm and cold temperature responses is particularly challenging for reproductive processes.

Flowers of high-mountain species not only have to cope with large diurnal temperature oscillations but with cold spells, which can occur at any time during the growing season. During cold spells, plants are mostly covered with snow resulting in temperatures constantly around zero for several days [25,26,27,28,29]. Summer cold snaps with snowfall are sudden events. Hence, flowers may be pollinated around midday at mild temperatures and may be covered with snow a few hours later. It is not known whether these flowers continue their reproductive development or not.

A recent study has shown that the performance of the pollen of mountain plants is remarkably flexible over a wide temperature range [30]. Pollen adhesion was possible from −2 to 40 °C, pollen germination and tube growth from 0–35 °C. Fertilization, however, occurred in a narrower temperature range from 5 to 30–32 °C in most species. In that study, flowers were exposed to the respective target temperature immediately after pollination. At low temperatures, pollen germination and pollen tube growth was slow from the beginning and, except for one species (*Gentianella germanica*), fertilization did not occur within the maximum exposure time of 50 h. Thus, the question of whether fertilization in mountain plants is considerably delayed or does not take place at all below 5 °C has been raised. A failure to perform in the cold would mean that initiation of seed development is restricted to warmer periods and that flowers that go through a cool period after pollination are lost for reproduction.

The present follow-up study should reveal more details about sexual performance in the cold. In laboratory experiments, seven plant species (Table 1), which differ in their elevational distribution range, were exposed to temperature conditions as they occur during summer cold snaps in high-mountains. To this end, flowers were pollinated with allopollen, gradually cooled down to 0 °C (the temperature below the snow), and kept at this temperature for up to four days. Pollen performance including fertilization was analyzed at the end of the cooling-down phase, after exposure for varying intervals at 0 °C, and after the respective warm-up phase. By chance we succeeded in verifying the experiment for one of the study species during a cold snap with snowfall at the mountain site.

**Table 1:** Characteristics of the investigated species.

Mountain belt ^1^	Plant species	Geographical distribution	Vertical distribution (m a.s.l.) ^2^	Sampling site ^3^	Flowering time	Gender sequence	Min–max distance stigma–ovary (µm) ^4^
Subalpine–alpine	*Gentianella germanica* (Willd.) subsp. *germanica*	Alpine grasslands in Western and Central Europe	500–2,400 (2,700)	P	September–October	Adicho-gamous	3,007–4,782
Alpine	*Ranunculus alpestris* L.	European Mountains	1,700–2,800 (2,940)	H	June	Adicho-gamous	781–1,144
Alpine	*Saxifraga caesia* L.	European Mountains	1,500–3,000	H	July–August	Prot-androus	1,502–2,146
Alpine–nival	*Saxifraga moschata* Wulfen	Eurasian mountains	>1,800 (4,200)	H	June–July	Prot-androus	1,124–1,833
Subnival–nival	*Cerastium uniflorum* (Clairv.)	European Alps	2,000–3,400	S	July–August	Prot-androus	2,124–4,096
Subnival–nival	*Ranunculus glacialis* L.	Arctic, European Mountains	2,300–4,000 (4,275)	S	June–July	Adicho-gamous	1,257–1,917
Subnival–nival	*Saxifraga bryoides* L.	European Mountains	2,000–4,000 (4,200)	S	July–August	Prot-androus	2,250–3,001

^1^ Mountain belt in the European Alps: subnival = alpine-nival ecotone [31], nival = ice-free areas within the glacier zone; ^2^ Vertical distribution in the European Alps according to [32,33,34,35]; numbers in brackets give the highest documented localities in the Swiss Alps; ^3^ Sampling sites: P = Mt Patscherkofel (1,950 m a.s.l.), H = Mt Hafelekar (2,350 m a.s.l.), S = Stubai Glacier foreland (2,880 m a.s.l.); ^4^ Mean minimum and maximum distance between stigma and first ovules within the ovary; n = 12−32 carpels per species.

We addressed the following questions: (1) to what extent are sexual processes during anthesis impaired by low temperatures in high-mountain plants—are they decelerated, temporarily interrupted, or irreversibly impaired; (2) in the case of deceleration or interruption, do processes continue in the normal way when temperature conditions become more favorable again; and (3) if so, how long can sexual processes be delayed or interrupted and remain functional?

Both alpine and nival species repeatedly experience cold weather periods during the growing season. Thus, we assumed that progamic processes of both species groups tolerate temperatures around zero to a certain extent. However, since nival plants regularly have to cope with subzero night temperatures we expected them to perform better during cold snaps than alpine species.

## 2. Results

### 2.1. Simulation of a Cold Snap in the Laboratory

**Figure 1:**
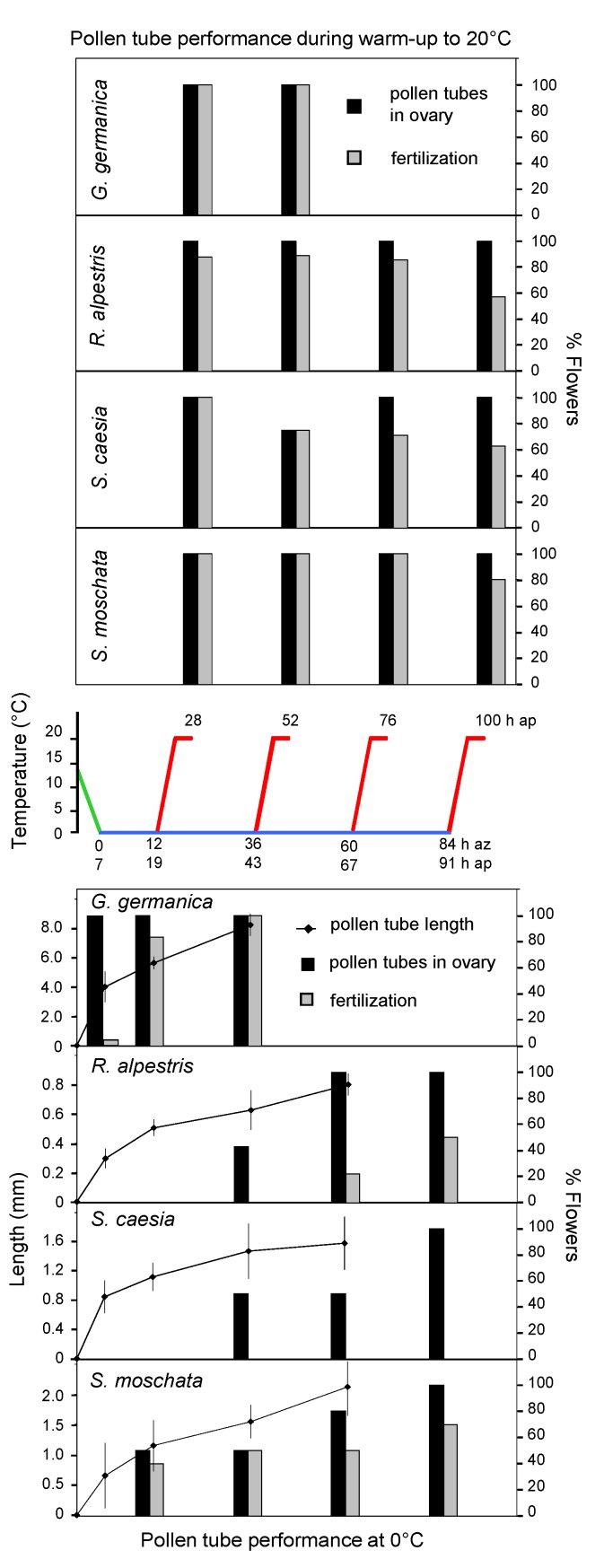
Performance of alpine species during the simulation of a cold snap in the laboratory. Middle graph: temperature course during the cooling-down phase (green line), the 0 °C-phase (blue line) and the warm-up phase (red line); numbers of the x-axis indicate the hours after pollination (ap) and the exposure times at zero (az). Lower graphs: mean lengths of pollen tubes (±SD) and percentages of investigated flowers with pollen tubes in the ovary and fertilized ovules, respectively, after the cooling-down phase (7 h ap) and after different exposure times at zero (12, 36, 60, 84 h az). Upper graphs: percentages of investigated flowers with pollen tubes in the ovary and fertilized ovules after different exposure times at time zero and the respective warm-up phase; *S. moschata* which occurs from the alpine to the nival zone was assigned to the alpine species group, as the investigated individuals originated from an alpine site.

**Figure 2:**
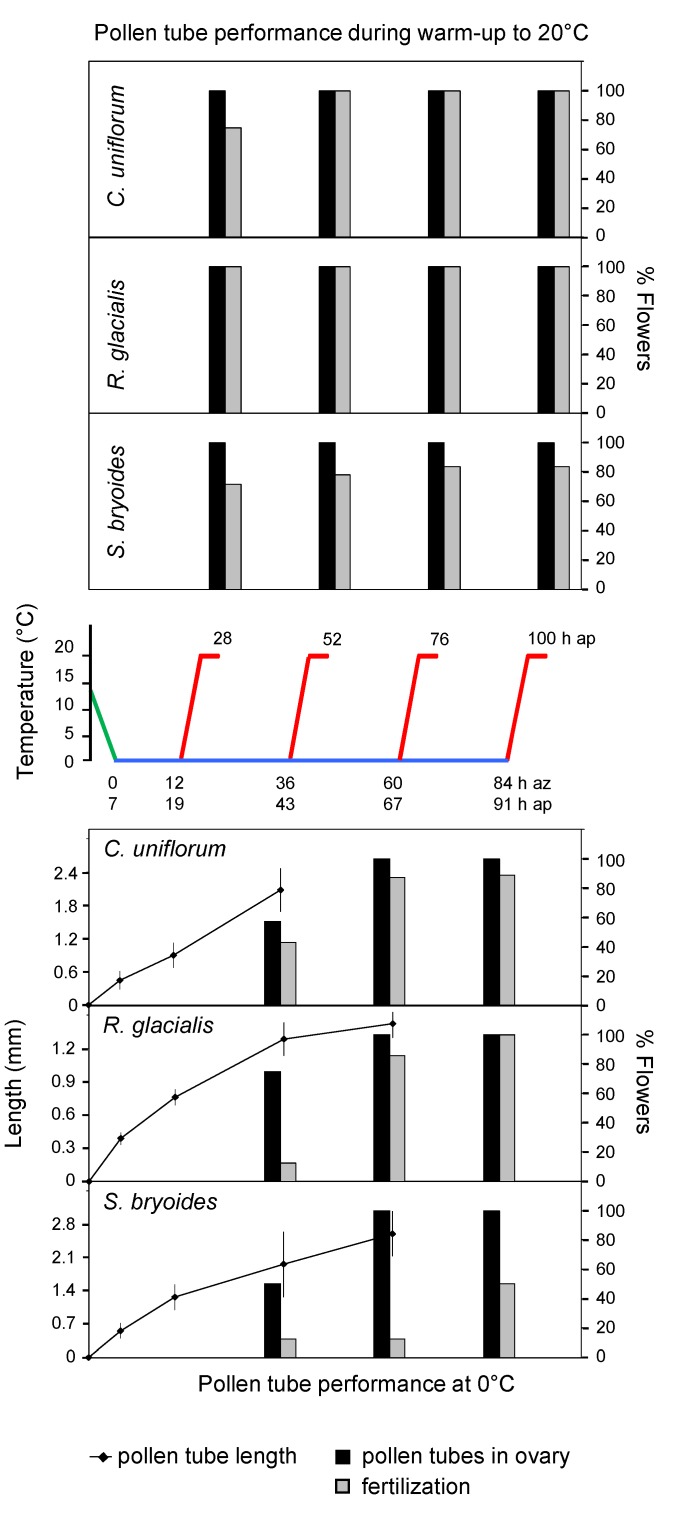
Performance of nival species during the simulation of a cold snap in the laboratory. For further details see Figure 1.

A cold snap simulation experiment consisted of a 7 h cooling-down phase from 14 to 0 °C, a cooling phase at 0 °C (12, 36, 60, 84 h, respectively), and a 5 h warm-up phase from 0 to 20 °C plus a further 4 h at 20 °C (cf. Figure 1 and Figure 2, middle graph). Immediately after pollination, plants were transferred to temperature-controlled freezers and the temperature run was started. During each temperature run pollen performance was checked after the cooling-down phase, after the respective cooling phase, and at the end of the 9 h warm phase.

Pollen germination and tube growth could be observed in all investigated flowers of all investigated species. Maximum percent pollen germination and tube growth were already attained during the 7 h cooling-down phase and did not change significantly during the cooling phase at 0 °C and the warm-up phase (Table 2). Percentage germination was above 70% in most species. The fraction of pollen grains that had germinated and continued tube growth into the style varied strongly among species and was between 17% (*G. germanica*) and 96% (*R. alpestris*).

**Table 2:** Pollen performance in the laboratory experiments and in the *in situ* experiment. Mean number ± SD of pollen grains per stigma lobe, germinated pollen grains and pollen tubes in the style after different phases. n carpels: number of investigated carpels per plant species and phase; n.d. not determined. As there were no significant differences among exposure times in the 0 °C and the warm-up phase (Kruskal–Wallis test) data of the respective phase were pooled together and a total average was calculated.

		Pollen performance after		
Laboratory experiments		cooling-down phase	0 °C-phase	warm-up phase
*G. germanica*	n grains	437 ± 44	379 ± 86	513 ± 93
	% germination	77 ± 11	78 ± 8	78 ± 7
	% tubes	18 ± 9	20 ± 12	17 ± 5
	n carpels	16	34	33
*R. alpestris*	n grains	6 ± 4	10 ± 5	11 ± 6
	% germination	97 ± 9	99 ± 3	99 ± 5
	% tubes	93 ± 11	96 ± 8	96 ± 8
	n carpels	50	156	151
*S. caesia*	n grains	147 ± 66	172 ± 64	188 ± 71
	% germination	67 ± 20	59 ± 22	62 ± 20
	% tubes	34 ± 14	31 ± 16	33 ± 19
	n carpels	19	62	64
*S. moschata*	n grains	227 ± 72	234 ± 123	256 ± 143
	% germination	79 ± 9	73 ± 28	76 ± 22
	% tubes	29 ± 13	37 ± 23	46 ± 19
	n carpels	16	62	74
*C. uniflorum*	n grains	36 ± 21	41 ± 27	35 ± 26
	% germination	84 ± 10	75 ± 19	70 ± 23
	% tubes	47 ± 15	39 ± 19	37 ± 18
	n carpels	64	126	102
*R. glacialis*	n grains	29 ± 13	n.d.	n.d.
	% germination	94 ± 6		
	% tubes	70 ± 12		
	n carpels	43		
*S. bryoides*	n grains	296 ± 105	282 ± 138	402 ± 157
	% germination	81 ± 11	70 ± 18	73 ± 19
	% tubes	47 ± 9	41 ± 13	41± 17
	n carpels	20	69	65
***In situ* experiment**			1st sampling	2nd sampling
*S. caesia*	n grains		234 ± 52	276 ± 86
	% germination		53 ± 19	54 ± 12
	% tubes		21 ± 11	18 ± 6
	n carpels		24	30

**Figure 3:**
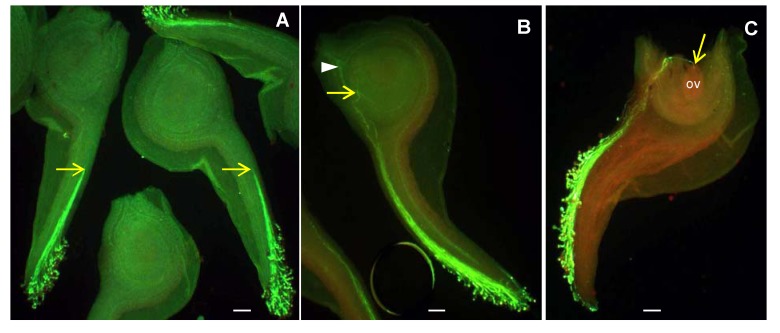
Pollen tube growth in *R. glacialis* during the cold snap experiment in the laboratory. (**A**) End of the cooling-down phase: Seven hours after pollination (ap) pollen tubes have passed about half of the length of the style; (**B**) 43 h ap (7 h cooling-down and 36 h at 0 °C): pollen tubes have entered the ovary; (**C**) 67 h ap (7 h cooling-down and 60 h at 0 °C): fertilized ovule. Yellow arrow: tip of the longest pollen tube; arrowhead: vascular strand; ov: ovule. Scale bars = 100 µm.

Pollen tubes grew quickest during the cooling-down phase. During the incubation at 0 °C tubes grew slower but elongated steadily. They entered the ovary and, except for in *S. caesia*, fertilizations occurred (Figure 1 and Figure 2, lower graphs; Figure 3). At zero, the mean growth rate was lower in the alpine species *R. alpestris* (10 µm·h^−1^) and *S. caesia* (19 µm·h^−1^) than in the nival species *C. uniflorum* (43 µm·h^−1^), *R. glacialis* (26 µm·h^−1^) and *S. bryoides* (38 µm·h^−1^), and the alpine-nival species *S. moschata* (27 µm·h^−1^). However, the time required until pollen tubes reached the ovary additionally depended on the species-specific style length (cf. Table 1). In *G. germanica*, due to a particularly high tube growth rate (121 µm·h^−1^), pollen tubes were already found in all ovaries after the cooling down phase; in one ovary fertilization had taken place. After 12 h at 0 °C, 80% of ovaries contained fertilized ovules. In *S. moschata*, 50% of ovaries showed pollen tubes, and about 40% contained fertilized ovules at the same point in time. In the remainder of the species, pollen tubes had reached the ovary within 36 h (50–80% of the flowers). Fertilization rate increased continuously during the exposure at time zero: at the end of the longest incubation period (84 h) about 50% (*R. alpestris*, *S. bryoides*), 70% (*S. moschata*), 90% (*C. uniflorum*) up to 100% (*R. glacialis*) of flowers showed fertilized ovules. During the warm-up phase, fertilization continued and was observed in 80–100% of flowers in the majority of species (Figure 1 and Figure 2, upper graphs). Exceptions were *R. alpestris* and *S. caesia* where the final flower fertilization rate at the end of the warm-up phase decreased with increasing duration of the preceding cooling phase.

Because of the squash-technique it was not possible to determine the exact fraction of fertilized ovules per ovary. At 0 °C only a smaller part of ovules became fertilized. In most species a number of pollen tubes grew along the placenta or past the micropyles without entering the ovules. After the warm-up phase fertilized ovules were clearly more frequent than at the end of the 0 °C-phase.

### 2.2. *In Situ* Experiment on S. caesia during a Summer Cold Snap with Snowfall

**Figure 4:**
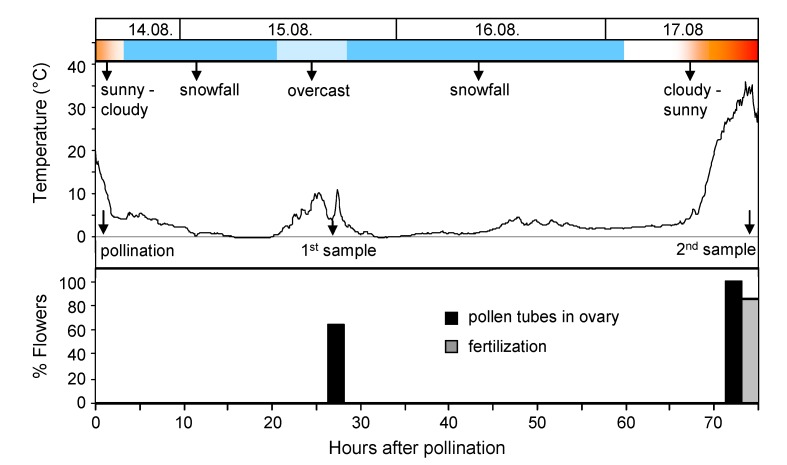
Weather conditions, temperature course and pollen performance in *S. caesia* during a cold snap at the alpine site (14–17 August 2005). Bars in the lower graph show the percentage of investigated flowers with pollen tubes in the ovary, and with fertilized ovules, respectively.

Figure 4 shows the course of temperature and weather conditions during a cold snap in the summer of 2005 at the alpine site. When on 14 August, between 1.00 and 2.15 pm, flowers of *S. caesia* were pollinated, the sun was shining and temperatures near the ground were around 17 °C. Shortly after temperatures dropped rapidly, it started to rain and finally to snow. Snowfall continued during the whole night and the following morning, and stopped in the afternoon for a few hours. At that time the first sampling took place. A further day with snowfall followed. On the fourth day the sky cleared up and temperatures rose again. The rest of the pollinated flowers was sampled in the early afternoon.

*In situ* pollen performance was largely consistent with the findings in the laboratory experiments (Table 2). Percent germination did not differ significantly between laboratory and field, only percent tube growth was lower at the natural site (*p* < 0.001, Mann-Whitney-U-test). In all investigated flowers, pollen germination and tube growth had taken place. In the first sample, taken on the second day of the cool weather phase, more than 60% of flowers showed pollen tubes in the ovary but no fertilization. In the second sample, drawn after rewarming, pollen tubes were found in all ovaries and fertilized ovules in more than 80% of ovaries (Figure 4). Germination and tube growth rates were not significantly different between the first and second sampling (Mann-Whitney-U-test). 

## 3. Discussion

### 3.1. Pollen Performance and Female Functions in the Cold

The most important finding of this study is that sexual functions of mountain plants remain largely intact even when pollinated flowers are exposed to near-freezing temperatures for several days. This can be seen as an adaptation to cool environments where a sudden fall in temperature with snowfall is possible at any time. The exact timing of pollen receipt and dispersal, and a quick start to the progamic processes are critical for successful reproduction in a stochastic climate. In insect pollinated species, the timing of these anthesis functions is largely coupled to pollinator activity. Visitation rates linearly increase with temperature and irradiation and are highest around midday [36,37,38,39]. Cool temperatures but also high wind speeds and high air humidity due to fog or rain reduce pollinator flight [39,40]. Flower and anther opening are genetically regulated [41], but additionally depend on appropriate environmental conditions. In accordance with pollinator activity, anthesis is enhanced by warm and dry conditions, and delayed or inhibited at high relative humidity and low temperatures (e.g., [42,43,44,45,46]). Thus, there is a high probability that in mountain plants pollination and the subsequent critical phases such as pollen adhesion (with complex recognition reactions), pollen hydration and pollen germination take place during favorable weather conditions.

From a recent study [30] we know that pollen grains of the study species germinate shortly after transfer to the stigma and grow pollen tubes into the style within a few hours. Pollen tubes protected by the tissues of the style and the ovary seem to be less vulnerable to unfavorable weather conditions. As shown by the field experiment on *S. caesia*, tube growth still proceeded when it was raining and snowing. At zero degrees, pollen tubes grew slowly but steadily which signifies that the continuous exchange of signals between the pollen tube and the transmitting tissue [47] can still function and structural alterations such as depolymerization of the actin cytoskeleton [48] obviously do not take place in mountain plants in the cold. Thus, the slow tube growth can be mainly seen as the result of a reversible retardation of metabolic processes. 

Penetrating the ovule and fertilization seem to be more critical phases. Though pollen tubes appeared sooner or later in the ovaries of all flowers, fertilization was less frequent or did not take place at all (*S. caesia*) when cooled. Pollen tubes are guided by signals coming from the unfertilized ovules as soon as they enter the ovary [49,50]. Obviously signals were often too weak in the cold so that pollen tubes grew past the micropyles without entering the ovules. However, these malfunctions were partly reversible as evidenced by the increase in fertilization during the warm-up phase. This became particularly apparent in *S. caesia*: both in the laboratory experiments and in the field experiment fertilization failed at temperatures near zero but recovered as soon as the temperatures rose. 

To survive a multiday period below the snow, flowers need to have a sufficiently long lifetime. Flowers of high-mountain plants generally live longer than lowland flowers to compensate for unreliable pollinator visits [51,52,53,54,55,56]. Flower longevity is not a strictly fixed trait but plastic to some extent in response to short-term environmental variation (e.g., [57,58,59,60]). The duration of pistil receptivity is critical for the female reproductive success of a flower. A separate study on the species being examined here revealed that within the different pistil functions, ovule receptivity is the first function to cease [56]. In most species, ovule receptivity began to decrease around day 10 after onset of anthesis. Thus, the progamic phase should be extendable for several days without loss of function. Some species such as *C. uniflorum* and *S. bryoides*, however, already lose ovule receptivity from day 4 on which means that in these cases a delay or interruption of progamic processes would only be tolerated for a short period of time. 

### 3.2. Comparison between Alpine and Nival Species

Since nival plants more often experience temperatures around zero during anthesis and are covered with snow for a longer period during cold spells (see chapter Site Temperatures in the Experimental Section), they were expected to perform better in the cold than species restricted to the alpine zone. This has proven true in part. Pollen tubes generally grew faster in nival species than in alpine species, however, the time span between pollination and the entry of the pollen tubes into the ovary depended on both the length of the style and the speed of pollen tube growth. Processes around fertilization seem to be less susceptible to near-freezing temperatures in nival species than in their alpine counterparts. In the nival species *R. glacialis* and *C. uniflorum*, and in the alpine-nival species *S. moschata*, fertilization occurred soon after the pollen tubes had arrived in the ovary. The proportion of fertilized flowers increased during exposure to 0 °C and reached values of up to 100%. In contrast, fertilization was strongly delayed in the alpine species *R. alpestris* and was absent in *S. caesia*. The longer the sexual processes were interrupted in the cold the lower was the fertilization frequency in the following warm-up phase. This suggests irreversible dysfunctions in the fertilization process, which would reduce the reproductive output during prolonged cold periods at least in these two alpine species. 

However there were exceptions both within the nival and the alpine species group. In the nival species *S. bryoides*, the fertilization rate was low and only reached 50% at zero degrees and 70–80% during the warm-up phase. The opposite applied to the alpine species *G. germanica,* which showed the shortest progamic phase of all investigated species. After 5 h at 0 °C, 80% of flower fertilization had taken place. These results are in line with the particularly short reproductive cycle of this species and a high seed output [61]. *G. germanica* flowers in several cohorts from August until late October when night frosts and snow falls occur more often and thus is obviously adapted to reproduce at low temperatures. 

### 3.3. Functional Limits for Progamic Processes in the Cold

What is the minimum temperature threshold for progamic processes to remain functional in mountain plants? At temperatures as low as −2 °C, Steinacher and Wagner [30] observed some pollen germination in all species investigated here, and even substantial tube growth in *G. germanica* and *R. glacialis*. Single experiments on *R. glacialis* at −3 °C have shown that the lower temperature limit of pollen activity was reached when freezing in reproductive tissues set in. From a recent study we know that flowers during anthesis are ice-sensitive in most species [27,62]. First frost damage (LT_10_) to the most susceptible reproductive structures (stigma, style, flower stalks) occurred between −2 and −4 °C (cooling-down rate 2 K·h^−1^, exposure time at target temperatures 4 h). Due to supercooling, however, flowers may cool down below temperatures causing frost damage. For the cushion plants *S. bryoides*, *S. caesia*, *S. moschata* and *Silene acaulis*, Hacker *et al*. [63] could show that ice nucleations occur independently in each single reproductive shoot—mainly in the stalks, and less frequently in the flower buds and flowers—and ice does not propagate into neighboring shoots. Independent freezing events limited to single reproductive shoots increase the chance of supercooling and thus the chance of survival for the remaining shoots and flowers. During anthesis the mean temperature range between first (LT_10_) and severe frost damage (LT_90_) was around 4 K in *R. glacialis*, *S. moschata* and *S. bryoides* but only 1.4 K in *S. caesia* and 2.8 K in *C. uniflorum* [27]. Injury following ice formation in reproductive shoots usually led to full fruit loss whereas reproductive success of frost-treated but undamaged shoots did not differ significantly from control values. This would suggest that shorter periods below zero during anthesis do not cause negative aftereffects on reproductive processes as long as ice does not form in reproductive structures. An exception might be *R. glacialis* whose reproductive shoots, in contrast to the other species, are ice tolerant. Ice spreads throughout at temperatures as high as −3 °C; however, frost damage mostly occurs at distinctly lower freezing temperatures (LT_10_ −6.2 ± 1.9 °C; [27]) possibly because of freeze-dehydration. As indicated above, extracellular ice formation within the flowers stops pollen tube growth but obviously does not impair reproductive structures as long as temperatures do not drop below the damage threshold. Thus, in this cold adapted plant species, reproductive processes are assumed to continue as soon as temperatures rise again even after extracellular ice has formed in the reproductive tissues. Further investigations are necessary to confirm this. 

## 4. Experimental Section

### 4.1. Study Species and Sampling Sites

The study species and their characteristics are summarized in Table 1. All species are hermaphroditic and insect pollinated. Selection criteria were: (1) Species are representative of different elevational zones in the European Alps (subalpine, alpine, subnival, nival; zonation according to [64] with different temperature regimes during the growing season [25,27]; (2) species flower at different times (early, mid and late flowering), and (3) there is detailed knowledge about reproductive characteristics from earlier studies for all species [29,30,56,60,61,65,66,67].

For laboratory experiments, plant individuals were sampled at the timberline on Mt Patscherkofel (1,950 m a.s.l., 47°12'N, 11°27'E; *G. germanica*), in the alpine zone on Mt Hafelekar (2,350 m a.s.l., 47°18'N, 11°23'E; *R. alpestris*, *S. caesia*, *S. moschata*), and in the subnival zone in the forelands of the Stubai Glacier (2,880 m a.s.l., 46°59'N, 11°07'E, Stubai Alps; *C. uniflorum*, *R. glacialis*, *S. bryoides*) in the Tyrolean Alps (Austria). Plants were sampled in the bud stage shortly before onset of anthesis. Whole individuals (*G. germanica*, *R. glacialis*), individual groups (*R. alpestris*), or parts of cushions (saxifrages, *C. uniflorum*) were excavated with adhering root balls (n = about 50 individuals or individual groups per species). Plants were wrapped in moist filter paper and placed in a cooler box for immediate transport to the laboratory. The time from sampling to reaching the laboratory was one hour (alpine sites) and two hours (subnival site), respectively. Plants were potted in original soil and kept at about 20 °C for 6–8 h during the day and then placed in a growth chamber at 4–5 °C for the remainder of the 24 h period (photoperiod 16 h light/8 h dark). 

About 140 flowers per species were marked with small color-coded plastic rings (cut from drinking-straws) by day of corolla opening. To avoid uncontrolled self-pollination, *G. germanica*, *C. uniflorum* and the saxifrages were emasculated before the male phase started. In the *Ranunculus*-species, carpels were delimited from stamens with plastic rings, 5 mm in height. Flowers of protandrous species (see Table 1) were marked with a second color-coded ring on the day the stigma became fully unfolded (onset of the female phase). For each species a set of flowers remained undisturbed (no emasculation, no labeling); these flowers acted as pollen donors. 

### 4.2. Simulation of a Cold Snap in the Laboratory

The temperature-data recorded during summer cold snaps at the mountain sites (cf. Figure 4) was used to create temperature runs that imitated naturally occurring cold snaps in the laboratory. A temperature run consisted of a 7 h cooling-down phase from 14 to 0 °C (2 K per hour), a cooling phase of different duration (12, 36, 60 and 84 h) at 0 °C (which corresponds to the temperature below the snow), and a warm-up phase from 0 to 20 °C (4 K per hour) plus a further 4 h at 20 °C (cf. middle graph in Figure 1 and Figure 2). Temperature runs were conducted in freezers (Liebherr GT 2102 Economy, Lienz, Austria), which were modified in order to expose plant samples to controlled temperature runs. The control unit of the system included a programmable data-logger, which allowed independent temperature runs in each of the freezers (software program LabView, National Instruments, Austin, TX, USA). Air temperatures within the propagating boxes (see below) were monitored by NTC-sensors, exact flower temperatures were measured with fine thermocouples.

For each temperature run 10–12 individuals or individual groups (*R. alpestris*) with open flowers in the appropriate female stage were selected. From previous investigations on the same plant species [30,56] it was known that the whole pistil was most receptive 1 d (*C. uniflorum*), 1–2 d (*S. bryoides*), and 1–3 d (the remaining species) after onset of the female phase. During this period, stigma papillae appear translucent and fully turgid. Beforehand pollination with allopollen, stigmas were checked to ensure that they were free of pollen under a stereo zoom microscope at 40× magnification (Olympus SZH). Stamens with freshly dehisced anthers of at least five different individuals were collected using tweezers and mixed in Eppendorf tubes. Small stamen portions with the adhering pollen mixture were brushed lightly over the stigma lobes until the surface was uniformly coated. Three to five flowers were pollinated per individual or individual group (*R. alpestris*).

Immediately after pollination, plants were enclosed in propagating boxes (lined with moist filter paper) with a clear cover (ventilation slots open), and exposed to the simulated cold snaps in the freezers. Flowers were sampled after the cooling-down phase, after the respective exposure time at 0 °C and at the end of the warm-up phase. Each time a total of 10–15 flowers from the different individuals was taken and immediately fixed in FPA 50 (50% ethanol, formalin, propionic acid; 90:5:5). Each individual was used for only one single temperature run.

### 4.3. *In Situ* Experiment on S. caesia During a Summer Cold Snap with Snowfall

The *in situ* experiment was conducted in the alpine zone on Mt Hafelekar (2,334 m a.s.l.) on naturally growing *S. caesia* individuals. During a separate field experiment, studying pollen tube growth of high mountain plants at the natural sites, a sudden cold snap with snowfall gave the opportunity to investigate postpollination processes under adverse weather conditions. In advance of the cold snap, 150 flowers in 24 individuals of *S. caesia* had been emasculated and plants bagged with a highly transparent fine-mesh organza. When weather conditions changed, 30 emasculated flowers with receptive stigmas in 18 individuals were available for the natural experiment. Flowers were hand-pollinated with allopollen as indicated above and plants were bagged again. Temperatures dropped immediately after pollination; within a few hours it started to rain and later to snow (see Figure 4). The first sample (12 flowers from 12 individuals) was taken the day after (26 h after pollination) when temperatures were still low. A second sample (18 flowers from 18 individuals) was taken after 74 h, when the adverse weather period was over and plant temperatures were around 20 °C again. Sampled flowers were immediately fixed in FPA 50.

### 4.4. Microscopic Analysis

Pollen performance was analyzed following [30], using the fluorescence standard method with aniline-blue [68]: Pistils were washed twice in dist. water (1 h per wash), soaked in 8 N NaOH-solution at 60 °C (15 min: *Ranunculus*-species, *C. uniflorum*; 20 min: saxifrages, *G. germanica*), rinsed again twice in dist. water and stained for at least 2 h with 0.1% aniline-blue in Sörensen phosphate buffer, pH 8. Pistils were gently squashed and examined under a fluorescence microscope (Olympus BH2, excitation filter 405–435 nm). The number of pollen grains on the stigma, the number of germinated grains (pollen tube longer than the pollen grain diameter), and the number of pollen tubes in the transmitting tract of the style were counted. Lengths of the longest pollen tubes in each investigated style lobe were measured using either an ocular-micrometer or a camera with image analyzing software (ProgRes CF, Jenoptik, Jena, Germany). Additionally, the percentage of flowers with pollen tubes reaching the ovaries was assessed and ovules were checked for fertilization. For the number of investigated carpels see Table 2.

### 4.5. Site Temperatures

During the *in situ* experiment on *S. caesia* ambient temperatures at the height of the flowers were recorded at 5 min intervals using small temperature loggers (Tidbit, Onset, Bourne, MA, USA). In three individuals, loggers were mounted inside the organza bags in immediate proximity to the inflorescences.

To ascertain the number of cold snaps with snowfall during the summer months June–August and the number of days with snow cover each time, microenvironmental temperature data from the different sites—where the investigated plant species occured—were analyzed. Plant temperatures were recorded at hourly intervals between 2002 and 2009 at the alpine site (2,350 m a.s.l., Mt Hafelekar), the subnival site (2,880 m a.s.l., Stubai Glacier), and a nival site (3,450 m a.s.l., Mt Brunnenkogel, 46°55'N, 10°52'E) using small Tidbit temperature loggers. Loggers were placed near the ground in plant cushions or below the leaves of *R. glacialis*. During the main growing season (June, July, August) two–four cold snaps occurred. On average flat growing plants were covered with snow for 2.7 ± 0.8 d in the alpine zone, for 3.1 ± 1.5 d in the subnival zone, and for 4.8 ± 3.7 d in the nival zone.

### 4.6. Statistics

For each species, mean percent pollen germination and pollen tube growth at different times of a temperature run in the laboratory were tested for statistical difference. As data were mostly not normally distributed, nonparametric tests (Kruskal–Wallis-test, Mann–Whitney-U-test) were applied. Analyses were performed with SPSS (SPSS Inc., Chicago, IL, USA), the critical level of significance α = 0.05.
